# A 4D continuous representation of myocardial velocity fields from tissue phase mapping magnetic resonance imaging

**DOI:** 10.1371/journal.pone.0247826

**Published:** 2021-03-01

**Authors:** Bård A. Bendiksen, Gary McGinley, Ivar Sjaastad, Lili Zhang, Emil K. S. Espe

**Affiliations:** 1 Institute for Experimental Medical Research, University of Oslo and Oslo University Hospital, Oslo, Norway; 2 KG Jebsen Center for Cardiac Research, University of Oslo, Oslo, Norway; 3 Bjørknes University College, Oslo, Norway; Henry Ford Health System, UNITED STATES

## Abstract

Myocardial velocities carry important diagnostic information in a range of cardiac diseases, and play an important role in diagnosing and grading left ventricular diastolic dysfunction. Tissue Phase Mapping (TPM) Magnetic Resonance Imaging (MRI) enables discrete sampling of the myocardium’s underlying smooth and continuous velocity field. This paper presents a post-processing framework for constructing a spatially and temporally smooth and continuous representation of the myocardium’s velocity field from TPM data. In the proposed scheme, the velocity field is represented through either linear or cubic B-spline basis functions. The framework facilitates both interpolation and noise reducing approximation. As a proof-of-concept, the framework was evaluated using artificially noisy (i.e., synthetic) velocity fields created by adding different levels of noise to an original TPM data. The framework’s ability to restore the original velocity field was investigated using Bland-Altman statistics. Moreover, we calculated myocardial material point trajectories through temporal integration of the original and synthetic fields. The effect of noise reduction on the calculated trajectories was investigated by assessing the distance between the start and end position of material points after one complete cardiac cycle (end point error). We found that the Bland-Altman limits of agreement between the original and the synthetic velocity fields were reduced after application of the framework. Furthermore, the integrated trajectories exhibited consistently lower end point error. These results suggest that the proposed method generates a realistic continuous representation of myocardial velocity fields from noisy and discrete TPM data. Linear B-splines resulted in narrower limits of agreement between the original and synthetic fields, compared to Cubic B-splines. The end point errors were also consistently lower for Linear B-splines than for cubic. Linear B-splines therefore appear to be more suitable for TPM data.

## Introduction

Measures of regional myocardial function play a key role in pre-clinical studies that aim to identify and describe mechanisms driving heart failure development, and to discover efficient targets for therapy. Myocardial velocities reflect myocardial function, and has been shown to carry important diagnostic information in a range of cardiac diseases [1–3]. They play a particularly important role in diagnosing and grading left ventricular diastolic dysfunction [4]. Velocity encoded tissue phase mapping (TPM) MRI enables measurement of myocardial velocities with high spatial and temporal resolution [5]. TPM data can also serve as a basis for deriving several characteristics that describes cardiac mechanical function on a regional level; such as strain [6, 7], fiber shortening [8], strain rate [9, 10], and work [11]. To derive these parameters, the velocity field must undergo either spatial differentiation (to get strain rate) or temporal integration (to get myocardial material point trajectories).

The velocity field measured by TPM is intrinsically a discretely sampled representation of the true underlying continuous velocity field. This constitutes a fundamental problem for integration of material point trajectories, as this in principle requires a continuous representation of the velocity field. The simplest solution to a lack of such a representation is to employ the nearest-neighbor assumption during integration [7]. In this approach the velocity at any location is assumed to be equal to the velocity in the closest voxel, i.e. the field is assumed to be piecewise (voxel wise) constant. The actual velocity of the myocardium, however, varies smoothly between imaging voxels, and any approximation of the velocity field is a potential source of error in estimation of functional parameters derived from TPM data. In principle, the validity of the nearest-neighbor approach improves with increasing image resolution. Increasing the image resolution will, however, prolong the scan duration and reduce the velocity to noise ratio of the data [12]. In velocity measurements, noise accumulates during integration of material point trajectories for assessment of strain [13, 14], and amplifies during strain rate calculations as a result of spatial differentiation [9]. B-spline processing has the potential to generate smooth and continuous vector fields based on discrete and noisy data [15]. Representing vector fields using B-splines also guarantees well-defined analytical differentiation [16]. B-spline processing has previously been used to analyze ventricular deformation from echocardiography [17], MRI tagging data [18], and to resolve the underlying tensor field describing biological fiber structure measured using Diffusion Tensor Imaging [19]. 3D biological fiber structure can be derived from Diffusion Tensor Imaging data through integration of seed points in the primary eigenvector field of the diffusion tensor field [20]. This analysis resembles the way deformation curves are derived from TPM data. We therefore hypothesized that B-spline processing is a suitable candidate for TPM applications.

In the present study, we developed a B-spline processing framework that is able to interpolate or approximate the measured time dependent velocity field of the hearts left ventricle (LV) measured with TPM-MRI. The proposed framework was developed to facilitate mathematically well-defined evaluation of the velocity field, while simultaneously reducing the influence of imaging noise on the field analysis. The suitability of both linear B-spline functions (LBS) and cubic B-spline functions (CBS) were tested. The proposed framework allows for individual levels of both temporal and spatial noise attenuation.

## Methods

### Continuous approximation of the velocity field

The theoretical basis for constructing continuous representations of discrete vector fields using B-splines has previously been outlined by Aldroubi and Basser [15]. Our implementation follows a similar approach to the approximation of continuous tensor fields commonly used in the processing of DTI-data [19]. Although most of the following concepts will be introduced for one-dimensional problems, we apply them to higher dimensions through the use of tensor product splines [21].

B-splines refer to a family of piecewise polynomial functions, of different polynomial order, that are commonly used to represent digital signals through either interpolation or approximation. In situations when the sampled signal of interest contains noise it can be an advantage to approximate the signal, rather than interpolate it. Unser and co-workers have previously suggested a scale conversion algorithm for the enlargement or reduction of digital images by arbitrary rational scale conversion factors [21]. In the scale conversion approach, the approximated signal is related to the B-spline basis through:

$$s_{\Delta }^{n}\left(x\right)=\overset{\infty }{\underset{k=-\infty }{\sum }}c_{\Delta }\left[k\right]\beta^{n}\left(x/\Delta -k\right)$$ (1)

**Fig 1:**
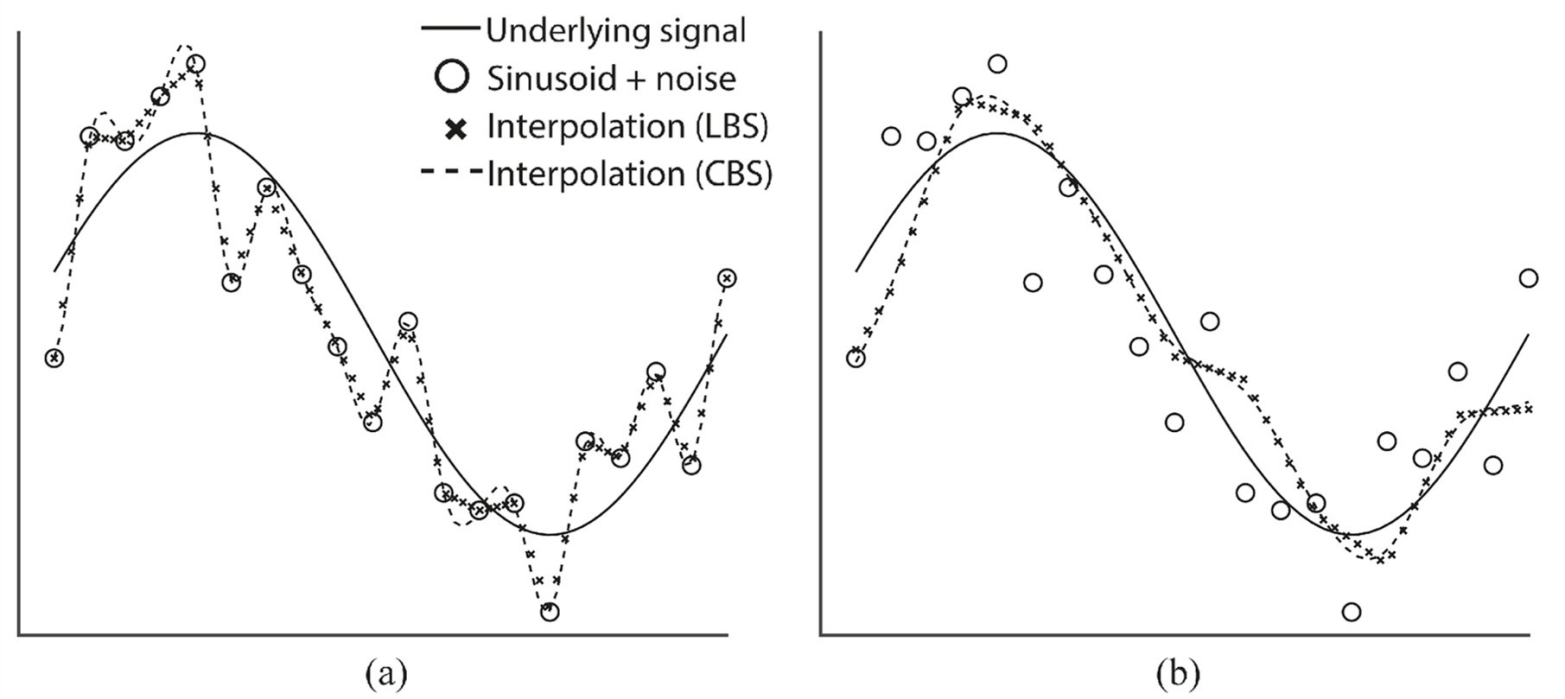
B-spline interpolation and approximation. a) Interpolation of a sampled, noise-contaminated sinusoid, using LBS (crosses) and CBS (dashed line). The continuous, noise free sinusoid is shown as a solid line b) Approximation a sampled, noise-contaminated sinusoid, using LBS (crosses) and CBS (dashed line).

Here, Δ represents the scale conversion factor, $s_{\Delta }^{n}$ represents the approximation of the signal, *β*^*n*^ represent a B-spline function of order *n*, and *c*_*Δ*_ represent a set of spline coefficients calculated by the algorithm. A scale conversion factor Δ = 1 corresponds to signal interpolation and Δ>1 result in signal approximation. A general approach for determining the optimal values of *c*_*Δ*_*[k]* in a way that minimizes the error in the least squares sense has previously been outlined by Unser *et al*. [21]. The difference between spline interpolation, and approximation using the scale conversion algorithm is illustrated Fig 1A and 1B, where the schemes have been applied on a noisy sample of a sinusoid, using both LBS and CBS.

In the proposed framework, we interpolate or approximate the components of the velocity field individually, using the following tensor product spline of *n*th order spline functions:

$$v_{\Delta ,q}^{n}\left(x,y,z,t\right)=\underset{i,j,k,l}{\sum }c_{\Delta ,q}\left[i,j,k,l\right]∙\beta^{n}\left(x/\Delta_{x}-i\right)∙\beta^{n}\left(y/\Delta_{y}-j\right)∙\beta^{n}\left(z/\Delta_{z}-k\right)∙\beta^{n}\left(t/\Delta_{t}-l\right)$$ (2)

where the superscript, *n*, denotes the spline order, and the subscript, *q*, denotes the *x*, *y*, and *z* component of the velocity field. For signals with more than one dimensions, the spline coefficients can be found by successive application of the one dimensional scheme along each dimension [21].

### Calculation of material point trajectories

Myocardial material point trajectories, ***r***(*t*), can be used to describe the position of a small volume of myocardium as a function of time, *t*. The trajectory is related to the velocity field of the material through integration of the following system of differential equations in time [13]

$$\frac{dr\left(t\right)}{dt}=v\left(r,t\right)$$ (3)

subjected to the initial condition ***r***(0) = ***r***_0_, where ***r***_0_ is some user defined “seed point location". ***v***(***r***, *t*) represents the spatiotemporal velocity field of the material. However, Eq 3 cannot in general be solved analytically, and it was therefore solved numerically using a forward Euler integration scheme [22]. One material seed point was generated in the center of each voxel that contained LV myocardium at the onset of systole (*t = 0*). The 3D position of each material point was then approximated at subsequent time points, throughout the entire cardiac cycle.

### Animal preparation

The Wistar rat that was included in the present study was sham operated for a study on myocardial infarction, following the procedure outlined in [23]. Thus, no additional animals were used solely for the purpose of this study, in accordance with the goal of reducing the number of animals used in experimental research. Anesthesia was induced before operation, using O_2_ and 4.0–4.5% isoflurane. During operation, the rat was intubated and ventilated on a ventilator with O_2_ and 2.0–3.0% isoflurane. As postoperative analgesia, the rat was given Buprenorphine. 2–3 rats per cage were housed in a temperature-regulated room with a 12:12 h light-dark cycle, and access to food and water ad libitum. The animal was anesthetized during MRI scans six weeks after operation, according to the description below. One day after the MRI examination, anesthesia was induced using O_2_ and 4% isoflurane and the rat was sacrificed by heart excision. The animal was cared for according to the Norwegian Animal Welfare Act. The use of animals was approved by the Norwegian Animal Research Authority (FOTS ID 3284/10102), and conformed to the Guide for the Care and Use of Laboratory Animals published by the US National Institutes of Health and the European Convention for the Protection of Vertebrate Animals used for Experimental and Other Scientific Purposes (ETS no. 123).

### Acquisition

All MRI images were acquired on a 9.4T T/210 mm/ASR horizontal bore magnet (Agilent Technologies Inc., Santa Clara, CA, USA) with a volume transmit coil (inner diameter 72 mm) and a 4-channel phase array coil (Rapid Biomed GmbH, Rimpar, Germany). The animal was anesthetized by 4.0–4.5% isoflurane, and put into the MRI lying in a prone position. Anesthesia was maintained using a stream of medical oxygen with 1.5–2.5% isoflurane at a flow rate of 1 L/min. Heart rate, respiration rate and body temperature were closely monitored (SA Instruments, Inc., New York, USA) during the experiments. Temperature was kept as close to 37 ^o^C as possible by thermostat-regulated warm air. TPM datasets with 4x undersampling were acquired, following the acquisition approach outlined by McGinley *et al*. [24]. A stack of 11 slices covering the LV of the rat heart were acquired with the following parameters: TE = 2.3 ms; TR = 3.2 ms; field-of-view = 45x45 mm; matrix = 128x32; slice thickness = 1.5 mm; gap = 0.0 mm; flip angle = 7°; venc = 13.9 cm/s using nine-point balanced encoding [25]. The venc was chosen to cover the spectrum of expected velocities of the myocardium [25], and was the same for all acquisitions. The spatial resolution and field-of-view were chosen to avoid fold-over artifacts, provide enough extracardiac structures for the eddy current compensation [26], as well as to keep gradient duty cycle within acceptable limits. The acquisition time was 42 min in total for all 11 slices. The acquisitions were prospectively triggered by ECG and prospectively respiration gated, in the freely breathing animal. The undersampled data were reconstructed using compressed sensing as previously described [24]. The resulting data set consisted of 128x128 voxels for each velocity field component, and 58 timeframes covering the entire cardiac cycle, for each of the 11 slices.

### Approximation of velocity fields

The TPM-measured 3D velocity components were combined into a 4D matrix, where the first three dimensions represented the laboratory frame of reference, and the fourth dimension represents time. This dataset will be referred to as the “original velocity field”, and includes myocardial velocities of the entire LV (11 slices).

**Fig 2:**
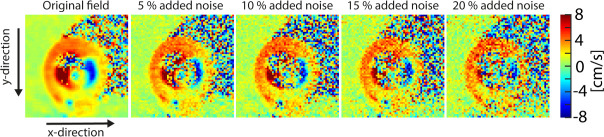
Velocity fields with varying levels of added noise. Illustration of the different levels of noise added to the velocity field. Here, the y-component of the field from a mid-ventricular slice early in systole is shown. The z-axis points in the through-plane direction.

The robustness of the approximation scheme towards imaging noise was tested on a set of “synthetic velocity fields”. Synthetic velocity fields with different levels of noise were generated by adding Gaussian noise to the real and imaginary components of the original MRI images, prior to velocity calculation. This was done to mimic TPM data with realistic noise profiles [27]. The added noise had a mean of 0, and standard deviations ranging from 5% to 20% of the 99th percentile signal intensity of the original images (Fig 2).

The LBS and CBS approximation schemes were applied on non-segmented velocity fields with different noise levels, using identical scale conversion factors in all three spatial dimensions. We used Δ_x_ = Δ_y_ = Δ_z_ = Δ_xyz_ = 1 for interpolation and the following fractions for approximation Δ_xyz_ = 60/58, 60/56, 60/54, 60/52 and 60/50. We chose to keep the temporal scale conversion factor fixed at 1 for all approximations, to avoid loss of rapid temporal changes in the velocity fields. In order to make the approximated fields comparable to the original fields, the approximated fields were reevaluated to provide discrete velocity fields with the original resolution.

The discrepancy between the original field and each of the synthetic fields was assessed by evaluating the voxel-wise difference in velocity of all voxels that included LV myocardium using Bland-Altman 95% limits of agreement (LoA). The voxels that included myocardium were identified by following the semi-automatic segmentation approach outlined by Espe *et al*. [28]. Briefly, end systolic and end diastolic masks are created based on manual delineation of the endocardium and epicardium. Material point trajectories are then calculated for seed points originating from within the manually segmented masks. These trajectories are used to identify the delineation of the myocardium at time points between the end diastole and end systole, and automatically remove seed points that transverses out of the manually segmented masks. Voxels that contained LV myocardium from all 11 slices and 58 timeframes were considered, giving in total 336620 myocardial voxels.

Spatial variations in the discrepancies were assessed by calculating residual fields (that is, the images of the voxel wise difference between the original and synthetic fields). In particular we wanted to investigate the effect of the discontinuities in the velocity field at the endocardial and epicardial borders on the approximation. First the residual fields were divided into an epicardial, a mid myocardial and an endocardial zone. Then the median and interquartile range of the residual fields were calculated for each zone individually, combining data from all time points.

### The effect of noise attenuation on material point trajectories

To isolate the noise attenuating effect of the framework on calculated material point trajectories, material points were propagated from their seed position by solving Eq 3, employing the nearest-neighbor method. For the calculation of material point trajectories, the myocardium was first segmented at the end diastolic and end systolic time point by manual delineation. Only seed points that originated from the myocardium at the end diastolic time frame were used for material point trajectory calculation. The trajectory for material points that were not located within the bounds of the LV mask at the end systolic time point were terminated, and the integration of that particular material point was flagged as unsuccessful, and disregarded [28].

A set of trajectories were calculated for the original velocity field and all the synthetic velocity fields, following the outlined scheme. The ability of the scheme to produce closed loops by simple forward integration was used as a measure of its accuracy. The trajectories should in principle form closed loops as the myocardium returns to its end-diastolic state. Therefore, the distance between the starting position and end position of each trajectory will be referred to as the “end point error” (EPE), defined as

$$\mathrm{EPE}=\|r_{\mathrm{end}\;\mathrm{position}}-r_{start\;position}\|$$ (4)

To ensure that any improvement in EPE was not mediated by an overall reduction in displacement, we also quantified the displacement of the material points from their initial position in end systole. This parameter will be referred to as the end systolic displacement (ESD), and was defined as

$$\mathrm{ESD}=\|r_{0}-r_{\mathrm{end}\;\mathrm{systole}}\|$$ (5)

**Fig 3:**
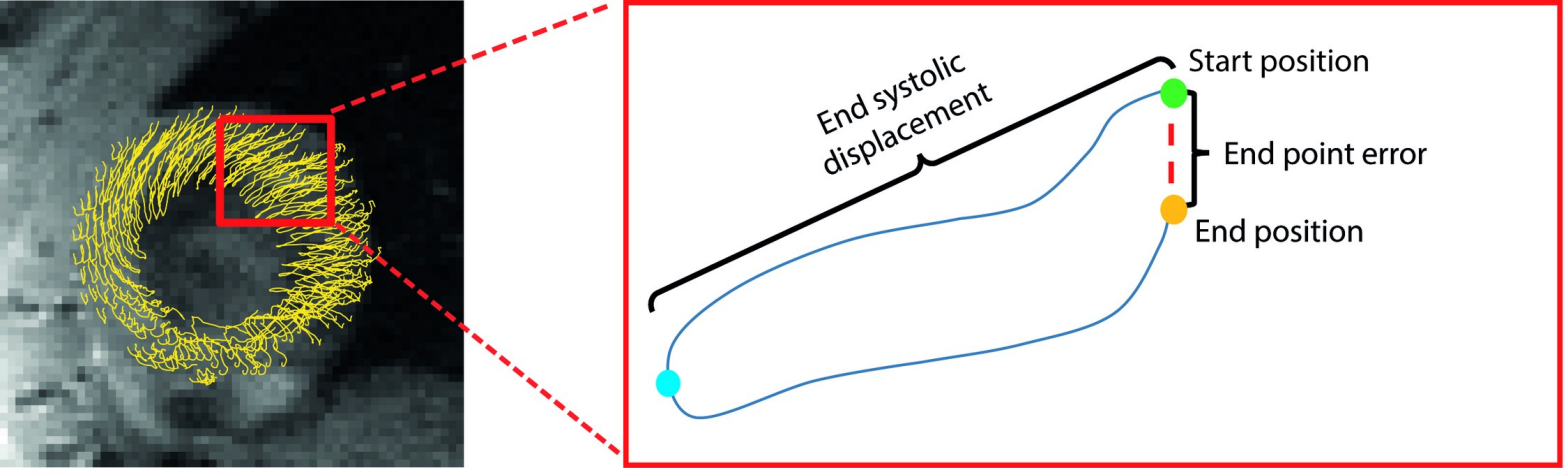
Material point trajectories with end systolic displacement and end point error. A graphical representation of the end point error, and end systolic displacement, for a hypothetical LV material point trajectory. For illustration purposes we show 2D projections of the estimated trajectories, displacements and EPEs, viewed from a mid-ventricular short axis.

Illustrations of the EPE and ESD are presented in Fig 3.

### Integration of trajectories in a continuous field

Next, we investigated the effect of integrating material points in a continuous field (piecewise linear or piecewise cubic) compared to integrating them in the measured, discrete, field (piecewise constant). First we interpolated the original field (with no added noise) using CBS and LBS. Afterwards, motion curves were integrated in the two continuous fields and the discrete field using a step size equal to the temporal sampling interval of the acquired TPM data [22]. The integration followed the scheme outlined above, in every other aspect. The accuracy of the trajectories was assessed by comparing the EPE-distributions from using the three different approaches.

### Data analysis and statistics

All post-processing and data analysis were performed in MATLAB 2018a (The MathWorks, Inc., USA). For computational efficiency, the images were cropped to 60x60 voxels per slice. In order to avoid discontinuities at the image borders, we chose to extend the image matrices on each side, in all dimensions, by using its mirror image [29].

## Results

### Approximation of velocity fields

**Fig 4:**
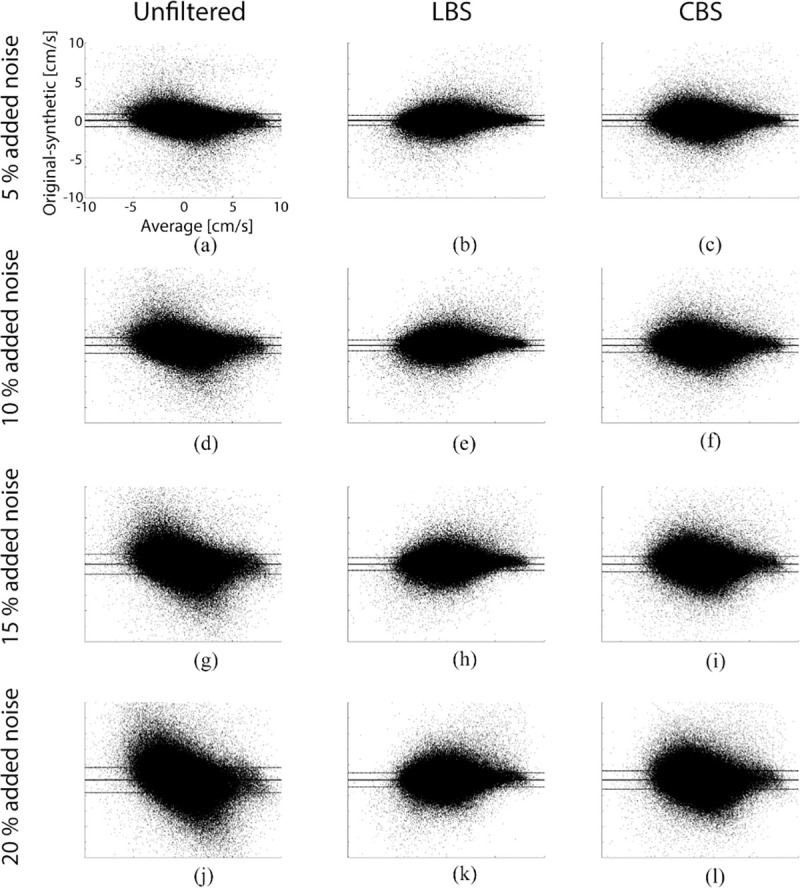
Bland-Altman analysis. Bland-Altman plots comparing the y-component of the original velocity field with different synthetic fields. Only Bland-Altman plots from processing with a scale conversion factor of Δ_xyz_ = 60/50 is shown.

The results from the Bland-Altman analysis of discrepancies between the original and synthetic velocity fields is summarized in Table 1. Representative Bland-Altman plots from the analysis is presented in Fig 4.

**Table 1:** Bland-Altman analysis.

Added noise:	5%	10%	15%	20%
	**x-component (in-plane)**			
**Unfiltered**	0.00 ± 0.80	0.0 ± 1.0	0.0 ± 1.3	0.0 ± 1.6
**LBS**				
**Δ_xyz_ = 60/58**	0.00 ± 0.56	0.00 ± 0.68	0.00 ± 0.86	0.0 ± 1.1
**Δ_xyz_ = 60/56**	0.01 ± 0.57	0.00 ± 0.65	0.00 ± 0.78	0.01 ± 0.94
**Δ_xyz_ = 60/54**	0.01 ± 0.56	0.00 ± 0.65	0.00 ± 0.77	0.00 ± 0.93
**Δ_xyz_ = 60/52**	0.01 ± 0.57	0.00 ± 0.64	0.00 ± 0.77	0.00 ± 0.92
**Δ_xyz_ = 60/50**	0.01 ± 0.64	0.00 ± 0.71	0.00 ± 0.82	0.00 ± 0.96
**CBS**				
**Δ_xyz_ = 60/58**	0.00 ± 0.71	0.00 ± 0.87	0.0 ± 1.1	0.0 ± 1.4
**Δ_xyz_ = 60/56**	0.01 ± 0.70	0.00 ± 0.83	0.0 ± 1.0	0.0 ± 1.3
**Δ_xyz_ = 60/54**	0.01 ± 0.69	0.00 ± 0.82	0.0 ± 1.0	0.0 ± 1.2
**Δ_xyz_ = 60/52**	0.00 ± 0.68	0.00 ± 0.81	0.00 ± 0.98	0.0 ± 1.2
**Δ_xyz_ = 60/50**	0.01 ± 0.77	0.00 ± 0.87	0.0 ± 1.0	0.0 ± 1.2
	**y-component (in-plane)**			
**Unfiltered**	0.00 ± 0.80	0.0 ± 1.0	0.0 ± 1.3	0.0 ± 1.6
**LBS**				
**Δ_xyz_ = 60/58**	0.00 ± 0.56	0.01 ± 0.69	0.01 ± 0.86	0.0 ± 1.1
**Δ_xyz_ = 60/56**	0.00 ± 0.57	0.01 ± 0.66	0.01 ± 0.78	0.01 ± 0.92
**Δ_xyz_ = 60/54**	0.00 ± 0.57	0.01 ± 0.65	0.01 ± 0.77	0.01 ± 0.90
**Δ_xyz_ = 60/52**	0.00 ± 0.56	0.01 ± 0.64	0.01 ± 0.76	0.01 ± 0.90
**Δ_xyz_ = 60/50**	0.00 ± 0.63	0.01 ± 0.69	0.01 ± 0.79	0.01 ± 0.92
**CBS**				
**Δ_xyz_ = 60/58**	0.00 ± 0.70	0.01 ± 0.87	0.0 ± 1.1	0.0 ± 1.4
**Δ_xyz_ = 60/56**	0.00 ± 0.70	0.01 ± 0.84	0.0 ± 1.0	0.0 ± 1.2
**Δ_xyz_ = 60/54**	0.00 ± 0.69	0.01 ± 0.82	0.0 ± 1.0	0.0 ± 1.2
**Δ_xyz_ = 60/52**	0.00 ± 0.68	0.01 ± 0.81	0.01 ± 0.98	0.0 ± 1.2
**Δ_xyz_ = 60/50**	0.00 ± 0.75	0.01 ± 0.85	0.0 ± 1.0	0.0 ± 1.2
	**z-component (through plane)**			
**Unfiltered**	0.00 ± 0.91	0.0 ± 1.2	0.0 ± 1.4	0.0 ± 1.7
**LBS**				
**Δ_xyz_ = 60/58**	0.00 ± 0.68	0.00 ± 0.79	0.00 ± 0.95	0.0 ± 1.1
**Δ_xyz_ = 60/56**	0.00 ± 0.66	0.00 ± 0.74	0.00 ± 0.86	0.0 ± 1.0
**Δ_xyz_ = 60/54**	0.00 ± 0.66	0.00 ± 0.73	0.00 ± 0.85	0.00 ± 0.98
**Δ_xyz_ = 60/52**	0.00 ± 0.66	0.00 ± 0.74	0.00 ± 0.85	0.00 ± 0.98
**Δ_xyz_ = 60/50**	0.00 ± 0.73	0.00 ± 0.79	0.00 ± 0.89	0.0 ± 1.0
**CBS**				
**Δ_xyz_ = 60/58**	0.00 ± 0.82	0.00 ± 0.97	0.0 ± 1.2	0.0 ± 1.4
**Δ_xyz_ = 60/56**	0.00 ± 0.81	0.00 ± 0.93	0.0 ± 1.1	0.0 ± 1.3
**Δ_xyz_ = 60/54**	0.00 ± 0.80	0.00 ± 0.91	0.0 ± 1.1	0.0 ± 1.3
**Δ_xyz_ = 60/52**	0.00 ± 0.79	0.00 ± 0.91	0.0 ± 1.1	0.0 ± 1.3
**Δ_xyz_ = 60/50**	0.00 ± 0.86	0.00 ± 0.95	0.0 ± 1.1	0.0 ± 1.3

Summary of the Bland-Altman analysis, where each component of the original velocity field and the different synthetic fields have been compared. Here we show the comparisons using both the LBS basis and the CBS basis, reported as (bias ± 95% LoA).

As expected, LoA between original and synthetic field grew wider with increasing noise. The Bland-Altman analysis did not reveal any systematic bias between the original and the synthetic noisy fields. Both LBS and CBS approximation resulted in narrower LoAs (Table 1).

**Fig 5:**
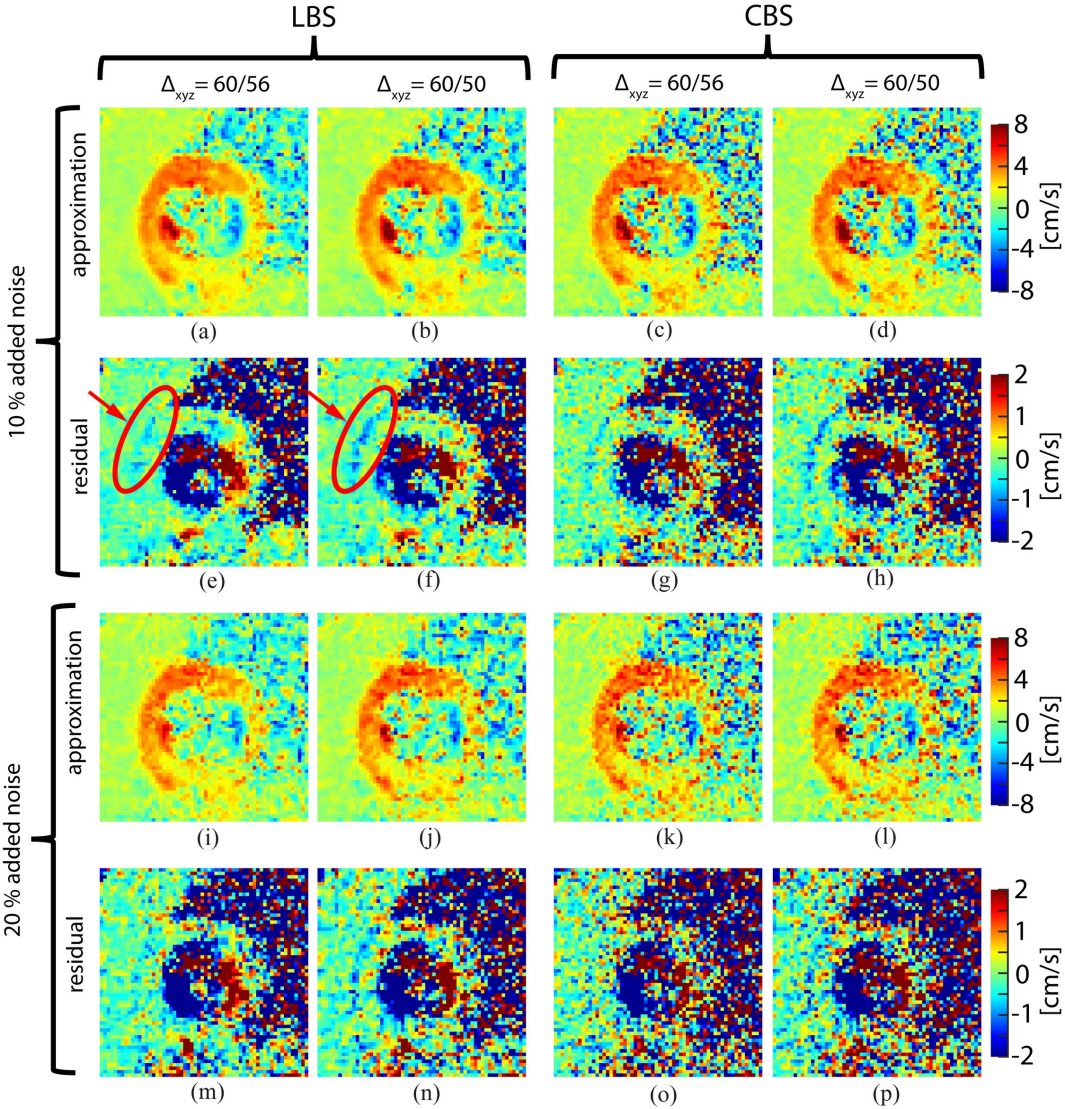
Field approximations and residual fields. Approximations of the y-component of the velocity field and their associated residual fields in early systole, with different B-spline basis, level of noise, and scale conversion factor. The section highlighted by the red circle illustrates discrepancies between the original and synthetic fields in the border between the LV anterior free wall and the chest wall.

The transmural variations in the LBS and CBS residual fields are reported in Table 2. We found that the interquartile range of the residual fields were larger in the endocardium and epicardium compared to the mid myocardial zone, indicating a less reliable field approximation in zones with discontinuous variations in velocity. The interquartile ranges grew with increasing noise level and increasing scale conversion factor. LBS resulted in consistently lower interquartile ranges than CBS. Some example images of residual fields are presented in Fig 5. In these example images only one time point early in systole is shown.

**Table 2:** Transmural analysis of residual fields.

		LBS				CBS			
		Δ_xyz_ = 60/56		Δ_xyz_ = 60/50		Δ_xyz_ = 60/56		Δ_xyz_ = 60/50	
		median	IQR	median	IQR	median	IQR	median	IQR
**x-component [cm/s]**									
**10%**	Endo	-0.03	0.74	-0.04	0.84	-0.04	1.03	-0.06	1.10
	Mid	0.04	0.65	0.01	0.72	0.00	0.94	0.01	0.99
	Epi	0.02	0.75	0.03	0.83	0.01	1.06	0.02	1.12
**20%**	Endo	-0.04	1.13	-0.05	1.20	-0.05	1.64	-0.07	1.63
	Mid	0.01	1.05	0.01	1.08	0.01	1.56	0.01	1.52
	Epi	0.02	1.17	0.04	1.19	0.02	1.70	0.03	1.64
**y-component [cm/s]**									
**10%**	Endo	-0.03	0.72	0.02	0.78	-0.02	1.02	-0.02	1.05
	Mid	-0.05	0.65	-0.05	0.70	-0.05	0.93	-0.05	0.96
	Epi	-0.06	0.75	-0.07	0.84	-0.07	1.05	-0.07	1.11
**20%**	Endo	-0.02	1.11	-0.02	1.15	-0.02	1.62	-0.02	1.57
	Mid	-0.04	1.04	-0.04	1.05	-0.04	1.55	-0.04	1.49
	Epi	-0.08	1.17	-0.10	1.20	-0.08	1.69	-0.10	1.64

Median and interquartile range of the epicardial, mid myocardial and endocardial residual fields of the in-plane velocity component, with different B-spline basis, level of noise, and scale conversion factor.

### The effect of noise attenuation on trajectories

**Fig 6:**
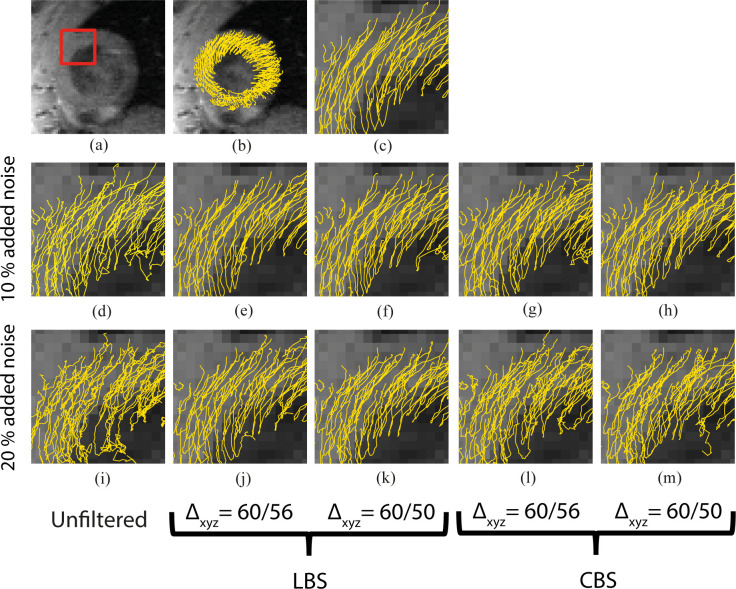
Material point trajectories. a) Anatomical short axis image of the heart at the onset of systole. The red box represents an arbitrary area of interest that has been used to compare trajectories between schemes. b) 2D projections of LV material point trajectories integrated based on the original velocity field, c) zoomed in view on the area of interest of the trajectories in (b), d-h) trajectories integrated in velocity field with 10% added noise with different B-spline basis functions and scale conversion factors (Δ_xyz_), and i-m) trajectories integrated in a velocity field with 20% added noise with different B-spline basis functions and scale conversion factors.

The noise attenuating effect of the framework on the calculated material point trajectories was assessed by propagating materiel points from their seed position by solving Eq 3, using the nearest-neighbor method for the original and synthetic velocity fields. Fig 6 allows a visual appreciation of selected representative material point trajectories. Visually, the trajectories integrated in the synthetically noisy fields (Fig 6D and 6I) appeared less smooth individually, and less coherent collectively, when compared to trajectories estimated from the original field (Fig 6C). Both CBS and LBS approximation appears to partially restore the smoothness and coherence (Fig 6D–6M).

**Fig 7:**
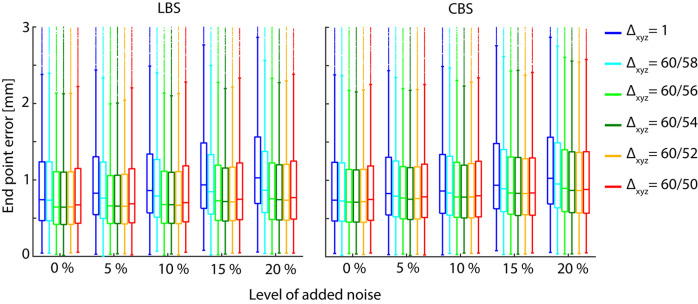
End point errors. End point error distributions of LV material point trajectories integrated based on the acquired- and synthetic velocity fields, representing different levels of imaging noise and choice of scale conversion (i.e. noise reduction), Δ_xyz_. All fields were integrated using the Euler method with nearest-neighbor interpolation. Here, Δ_xyz_ = 1, corresponds to an unfiltered data set. The central mark indicates the median, and the bottom and top edges of the box indicate the 25th and 75th percentiles, respectively. The whiskers extend to the most extreme data points not considered outliers by MATLAB’s boxplot function. The plot axes are scaled to show the body of the box plots more clearly, and do not display extreme outliers >3mm.

We found no systematic variation in number of successful trajectory integrations by varying the scale conversion factor. The distribution in EPE for the trajectories are presented in Fig 7. With increasing scale conversion factor, EPE assumes a convex shape for all the noise levels investigated. Furthermore, it appears that the scale conversion factor that minimizes the median EPE, simultaneously minimize the spread in EPE.

**Fig 8:**
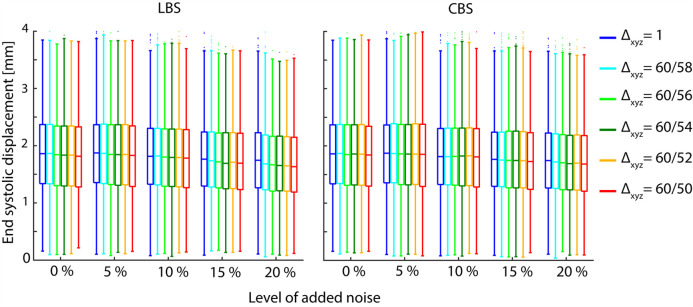
End systolic displacements. End systolic displacement distributions of LV material point trajectories integrated based on the acquired- and synthetic velocity fields, representing different levels of imaging noise and choice of scale conversion. The plot axes are scaled to show the body of the box plots more clearly, and do not display extreme outliers.

Increasing the noise intensity or scale conversion factor appears to reduce the median ESD (Fig 8).

### Integration of trajectories in a continuous field

**Fig 9:**
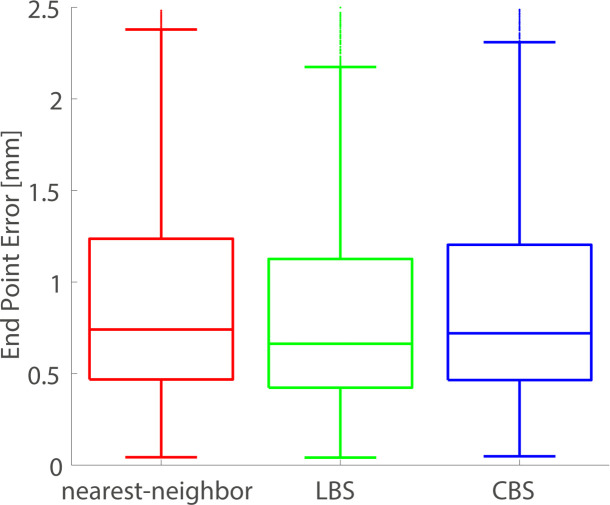
Accuracy of trajectories integrated in a continuous field. End point error distributions of LV material point trajectories integrated in a continuous representation of the field. Here we compare nearest-neighbor interpolation, LBS interpolation, and CBS interpolation. The trajectories were integrated with the Euler method, using a step size equal to the temporal sampling interval of the acquired TPM data.

A comparison of the EPE-distributions for trajectories integrated in a continuous field (piecewise linear or piecewise cubic) and the original discrete field (piecewise constant) is presented in Fig 9. The LBS interpolation yielded a narrower EPE-distribution, with a lower median, compared to the nearest-neighbor interpolation. The CBS interpolation gave similar results as the nearest-neighbor interpolation.

## Discussion

### Summary of results

We have developed a framework that 1) recovers a smooth and continuous approximation of a velocity field from noisy TPM data and 2) allow analytical, rather than numerical, subsequent processing of the data. The smoothness of the approximated field can be readily adjusted by varying the scale conversion factor, Δ, in any of the field’s dimensions. This way, our framework can interpolate (for Δ = 1) or approximate (for Δ>1) the measured field using an either piecewise linear- or piecewise cubic B-spline scale conversion algorithm. We assessed the ability of the framework to recover a velocity field after addition of random noise, and found that the LoAs between the original and the synthetic fields were narrower after the application of the framework. We also calculated myocardial material point trajectories through temporal integration of the original and synthetic fields. Linear B-splines resulted in narrower limits of agreement between the original and synthetic fields, compared to Cubic B-splines. The end point errors in the calculated material point trajectories were also consistently lower for Linear B-splines than for cubic. Therefore, Linear B-splines appear more appropriate than cubic for TPM data.

### Approximation of velocity fields: Edge effects

B-spline processing reduced the influence of noise in the synthetic velocity fields (Fig 4). In general, the LoA exhibited an either declining or convex trend with increasing scale conversion factor (Table 1). The residual fields revealed a relatively larger discrepancy in the epicardial and endocardial borders of the LV compared to midmyocardial regions (Table 2). In effect, the scale conversion algorithm performs a compression of the velocity field for scale conversion factors larger than one (Δ>1). In order to minimize the loss of information upon compression, it expands the width of the basis functions. This leads to an increasing degree of averaging among voxels in the same neighborhood. Errors due to averaging are therefore expected to be more severe in regions where discontinuities in the velocity field occurs, such as at the endocardial and the epicardial borders of the LV. This is supported by our observation that this error became more apparent for larger scale conversion factors (Fig 5E and 5F). Such edge artifacts might introduce systematic errors in subsequent processing of the velocity fields, for instance when calculating strain, which is known to vary transmurally [30]. This challenge may be addressed by forcing the algorithm to discard data points outside the myocardium, and/or reject integrated motion paths that travels outside the myocardium [7].

### The effect of noise attenuation on trajectories

We show that the suggested framework improves the precision and accuracy of LV material point trajectories derived from noisy TPM data. Both the width and the median of the EPE distributions varies in a convex manner with increasing scale conversion factor (Fig 7). Interestingly, the same scale conversion factor minimized both the median and spread in the EPE for all levels of noise investigated, indicating that there exists an optimal scale conversion factor for a given set of TPM data. The decay in ESD (Fig 8) with increasing Δ does not appear to fit the convex trend in the EPE or the magnitude in the EPE reduction. We therefore do not believe that the reduction in EPE is mediated by a reduction in the overall movement of the material points.

### Comparison of LBS and CBS

The LBS approximations reduce the LoAs between the original and synthetic velocity fields more than the CBS scheme, for all investigated levels of noise (Table 1). Discrepancies between the original and synthetic fields near to the endocardial and epicardial borders were also consistently lower when LBS were used, compared to CBS (Table 2). When we compared the effect of noise attenuation on the integration of material point trajectories, the LBS-scheme yielded a lower EPE for all the tested scale conversion factors, and all the noise levels. Cubic B-spline approximation is intrinsically better suited to capture rapid oscillations in the input signal than LBS [21] and therefore more prone to conserve noise. In addition, it tends to introduce oscillations near large spatial variations in the dataset [21]. In TPM data, we do not expect the velocity field to oscillate between voxels.

We also compared trajectory integration using the nearest-neighbor interpolation to LBS and CBS interpolation. Interestingly, we observe little difference in EPE when we compare material point integration using nearest-neighbor interpolation and CBS interpolation. In contrast, LBS-interpolation yields an EPE-distribution with a lower median and narrower interquartile range. In general, higher order polynomial functions are more likely to overfit the data compared to lower order polynomials. On the other hand, lower order polynomials such as the pricewise constant polynomial used in the nearest-neighbor interpolation are more likely to underfit the data. The problem of overfitting can be remedied by approximating rather than interpolating, as demonstrated by comparing Fig 1A and 1B.

### Limitations

A limitation of the study is that we do not have a noiseless velocity field that realistically mimics the motion of the LV. We therefore do not know if the approximated field is closer to the ground truth, compared to the original field. This limitation extends to the effect of the framework on the calculated LV material point trajectories, where we instead use the ability of the scheme to produce closed trajectories to assess the validity of the method.

### Future perspectives

Although there are some differences in the contractile pattern between human and rat hearts, strains, the relative temporal resolution (frames per cardiac cycle) and the anatomical resolution (imaging resolution divided by heart size) are similar between rats and humans [31]. We therefore expect that the proposed framework is useful also for clinical data. This calls for further studies into clinical validation of the method.

The ratio between peak early diastolic mitral flow (E), and peak early diastolic mid ventricular myocardial velocities (e′) measured with echocardiography can be used to predict LV filling pressures [32]. E/e’ is one of the key biomarkers recommended for diagnosing diastolic dysfunction in the presence of normal LVEF [4]. In the presented mapping data, e’ can be extracted directly for any region of the left ventricle–due to the superior geometric control offered by MRI compared to echo. We have previously shown that also E can be measured in the rat heart using MRI [33]. The proposed post processing framework allows the velocity field to be represented analytically, rather than discreetly, thereby allowing the field to be evaluated at any point in space and time. It also improves the precision and accuracy of myocardial velocities, in the presence of imaging noise. We therefore believe that the proposed framework has the potential to improve the assessment of regional diastolic dysfunction using MRI.

Myocardial TPM data has also been shown to be valuable in detailed assessment of myocardial mechanical function, through calculation of strain and strain rate tensor fields [9, 14]. Calculation of strain and strain rates relies upon spatial differentiation of velocity and displacement fields, which is not well defined for discrete fields. Imaging noise further hampers this assessment, as noise is amplified by numerical differentiation [34]. Approximation of the velocity field through B-splines remedies this problem by attenuating the influence of noise, while simultaneously making the field susceptible to well defined analytical differentiation [16]. We therefore believe that the proposed framework is well suited for advancing the use of TPM for detailed assessment of myocardial mechanical function. It has the potential to be a valuable tool for detecting regional perturbations in cardiac function which is of great value in pre-clinical studies on heart failure mechanisms. In a clinical setting it could serve, not only serve as a more robust velocity assessment tool, but also as a pre-processing step for calculating strain or strain rate from TPM data. Future studies are warranted investigating the value of this framework in deriving parameters such as strain and strain rate. We also believe that the proposed framework has potential uses in similar applications of MRI, such as for the assessment of flow.

## Conclusion

In this study we have presented a B-spline framework for analyzing discrete myocardial velocity fields, acquired using TPM. To the best of our knowledge, we are the first to propose a post processing framework that can generate a smooth and continuous representation of myocardial velocities, from TPM data. We have shown that the framework reduces the impact of noise on the measured velocity fields. Furthermore, we show that it improves the robustness of material point trajectory integration in the presence of noise. Linear B-splines seem more suitable for describing myocardial motion than cubic B-splines.
